# Commentary: Boosting Vaccine-Elicited Respiratory Mucosal and Systemic COVID-19 Immunity in Mice With the Oral *Lactobacillus plantarum*

**DOI:** 10.3389/fnut.2022.846379

**Published:** 2022-02-18

**Authors:** Yongbo Kang, Yue Cai

**Affiliations:** Department of Microbiology and Immunology, School of Basic Medical Sciences, Shanxi Medical University, Taiyuan, China

**Keywords:** COVID-19, vaccine, gut microbiome, probiotics, prebiotics, synbiotics

## Introduction

We read with interest the article “*Boosting Vaccine-Elicited Respiratory Mucosal and Systemic COVID-19 Immunity in Mice With the Oral Lactobacillus plantarum*” (1). In this article, the authors demonstrated that probiotic *Lactobacillus plantarum* GUANKE (LPG) is able to boost severe acute respiratory syndrome coronavirus 2 (SARS-CoV-2)-vaccine-induced effective memory immune response by enhancing interferon signaling and suppressing apoptotic and inflammatory pathways. Co-administration of SARS-CoV-2 vaccine and LPG shows a great potential to improve coronavirus disease 2019 (COVID-19) vaccination efficacy. Routine administration of LPG is likely to enhance the host innate immune response to combat SARS-CoV-2. Altogether, the study not only contributes to the containment of the pandemic, but also gives us some inspiration for future research direction.

## Future Research Directions for Improving the Efficacy of SARS-CoV-2 Vaccination

Based on our view, it is vital to underscore the relationship between probiotics and efficacy of SARS-CoV-2 vaccination. Vaccination plays a key role in COVID-19 prevention and control. Nevertheless, studies have demonstrated that the protective effect of the vaccine decreases within 6 months after the second dose, especially in the elderly. With the emergence of breakthrough infections and new variants, there exists an urgent need for the enhanced immunity to strengthen and prolong the protective effects of the vaccine. In addition, vaccinating every 6 months or every year to improve protection becomes obviously a huge challenge and not the best public health strategy. As a result, improving the efficacy of SARS-CoV-2 vaccination is very essential and remains a hot research topic on containing the pandemic. Probiotics, prebiotics, or synbiotics may represent an efficient approach to increase the efficacy of SARS-CoV-2 vaccination (2). However, available data on this field remain limited, and relevant scientific studies had only begun recently. Thus, much more research is needed in the future. First, clinical studies with large sample sizes from different countries and regions are needed to clarify the relationship between gut microbiota and efficacy of SARS-CoV-2 vaccination. Second, it also needs to be clarified which types of gut microbiota can provide best efficacy for SARS-CoV-2 vaccination. Third, it is also necessary to determine which beneficial bacteria are closely related to improvement in the efficacy of SARS-CoV-2 vaccination. Furthermore, findings on these will help to identify some probiotics that may increase the efficacy of SARS-CoV-2 vaccination when used in combination. Last but not least, it is also very valuable to explore the mechanism of action of prebiotics or synbiotics in combination with SARS-CoV-2 vaccination.

## Discussion

All in all, based on the timely and meaningful research results of this article, we wish to draw the attention of readers to the relationship between gut microbiota and efficacy of SARS-CoV-2 vaccination, and which probiotics, prebiotics, or synbiotics can be used or have the potential to improve the efficacy of SARS-CoV-2 vaccination. Those will be possible to provide a new weapon for COVID-19 prevention and control. Finally, we hope that everyone will work together to defeat the pandemic at an early date.

## Author Contributions

YK collected the data and wrote most of the manuscript with help from YC. Both authors contributed to the article and approved the submitted version.

## Conflict of Interest

The authors declare that the research was conducted in the absence of any commercial or financial relationships that could be construed as a potential conflict of interest.

## Publisher's Note

All claims expressed in this article are solely those of the authors and do not necessarily represent those of their affiliated organizations, or those of the publisher, the editors and the reviewers. Any product that may be evaluated in this article, or claim that may be made by its manufacturer, is not guaranteed or endorsed by the publisher.
